# Using Supermarket Loyalty Card Data to Provide Personalised Advice to Help Reduce Saturated Fat Intake among Patients with Hypercholesterolemia: A Qualitative Study of Participants’ Experiences

**DOI:** 10.3390/nu13041146

**Published:** 2021-03-31

**Authors:** Charlotte L. Lee, Paul N. Aveyard, Susan A. Jebb, Carmen Piernas

**Affiliations:** 1 Nuffield Department of Primary Care Health Sciences, University of Oxford, Radcliffe Primary Care Building, Radcliffe Observatory Quarter, Woodstock Road, Oxford OX2 6GG, UK; charlotte.lee@phc.ox.ac.uk (C.L.L.); paul.aveyard@phc.ox.ac.uk (P.N.A.); susan.jebb@phc.ox.ac.uk (S.A.J.); 2 Oxford Biomedical Research Centre, National Institute for Health Research, Oxford OX2 6GG, UK

**Keywords:** saturated fat, LDL cholesterol, cardiovascular disease, grocery shopping, brief support, shopping feedback, primary care, qualitative

## Abstract

Background: The ‘Primary Care SHOPping Intervention for Cardiovascular Disease Prevention’ (PCSHOP) trial tested the effectiveness and feasibility of a behavioural intervention to reduce saturated fat in food purchases. The intervention offered feedback from data collected through a supermarket loyalty card to supplement brief advice from a nurse. This qualitative study aimed to describe participants’ experiences of receiving this intervention. Methods: We conducted semi-structured, one-to-one, telephone interviews with participants from the PCSHOP trial. Interviews were audio-recorded and transcribed verbatim. We employed the one sheet of paper technique and a thematic analysis to develop high-level themes in NVivo software. Results: Twenty-four participants were interviewed (mean age: 63 years (SD 12)). They reported that the brief advice did not provide any new information but they welcomed the sense of accountability the nurse provided. The personalised shopping feedback and healthier swap suggestions provided novel information that challenged previously held beliefs about the saturated fat content of food purchases and encouraged some positive dietary changes. However, the taste preferences of the participant or other household members were a barrier to changing food shopping behaviours. Conclusion: Harnessing loyalty card data is a novel and acceptable method to offering personalised dietary feedback. Yet, issues on the suitability of the healthier swap suggestions limited the extent of dietary change. Trial registration: ISRCTN14279335. Registered 1 September 2017.

## 1. Introduction

A diet high in saturated fat can increase low-density lipoprotein (LDL) cholesterol and risk of cardiovascular disease (CVD) [1,2]. Saturated fat intake is above recommendations in many countries. For example, it is 13% in the United Kingdom (UK) compared with guidelines advising no more than 10% [3,4]. A systematic review of randomised controlled trials (RCTs) showed that dietary advice designed to decrease consumption of high saturated fat foods can modestly reduce LDL cholesterol [5]. However, most of the successful interventions have involved high-intensity skilled support [6,7], which is not possible to implement for the population at large [8]. Indeed, public health educational campaigns have led to only small changes in saturated fat intake suggesting that new approaches are needed to achieve population-level targets.

Grocery stores typically account for 71% of weekly food purchases, and supermarket food purchases are highly correlated with food consumption, including high saturated fat foods like meat, dairy, and confectionary [9]. This means interventions that target grocery stores have a potential to reduce saturated fat intake. A systematic review of supermarket interventions identified healthier swap suggestions as a potentially effective strategy to improve the nutritional quality of the food purchasing [10], and a recent intervention in an online experimental supermarket confirmed healthier swaps helped reduce saturated fat in purchases [11]. Recent technological developments within large supermarket chains, such as loyalty card schemes where the customer scans their card for every purchase, allow for objective monitoring of food purchases. This could enable automated and personalised feedback to the individual, including providing personalised swaps for healthier foods at a scalable level [12]. To date, a systematic review has shown it is feasible to use loyalty card data from supermarkets to monitor food and nutrition purchases [13]. Yet, evidence on the acceptability and usefulness of using loyalty card data in a behavioural change intervention has not been previously explored.

The ‘Primary Care Shopping Intervention for Cardiovascular Disease Prevention’ (PCSHOP) RCT aimed to test the effectiveness and feasibility of a behavioural intervention to reduce high saturated fat food purchases. The intervention used data collected through a supermarket loyalty card to supplement brief advice from a healthcare practitioner (HCP) like a nurse, compared with no intervention. Details of the trial design and intervention groups are reported elsewhere [14,15]. Briefly, participants randomised to the ‘Brief Support’ (BS) group received a 10-min general counselling session with a nurse in primary care who advised participants about the effect of saturated fat on blood cholesterol and CVD risk and provided tips to reduce saturated fat. Participants randomised to ‘Brief Support plus Shopping Feedback’ (SF) group were additionally provided with monthly shopping reports and were encouraged to use the reports to guide their shopping and to monitor their progress in reducing saturated fat. The reports contained information on the weekly amount of saturated fat in their shopping, top five contributors to saturated fat, plus lower saturated swap suggestions.

The RCT showed the intervention groups decreased the percentage saturated fat based on supermarket purchases from baseline to follow-up, but these changes were not significantly different from the control group [15]. In this qualitative study, we aimed to understand in more detail the experiences of participants who were randomised to receive both brief advice from a nurse and personalised shopping feedback offering healthier swaps. In particular, our objectives were to understand the value participants attached to the brief advice, the usefulness of the shopping feedback, and how the intervention could be optimised to enhance the effectiveness for dietary change. The results of this study will provide evidence on the acceptability and usefulness of using loyalty card data to refine an automated and scalable intervention that could offer population-level health benefits.

## 2. Materials and Methods

We report this study following the Consolidate Criteria for Reporting Qualitative Studies (COREQ) criteria [16].

### 2.1. The PCSHOP Trial

PCSHOP was a parallel-group RCT that tested an intervention with a three-month follow-up conducted in four primary care practices in Oxfordshire, UK. Participants were individually randomised in a 1:3:3 ratio to either the control group or one of two active intervention groups. The trial was reviewed and approved by the National Health Service Health Research Authority (HRA) Research Ethics Committee (Ref: 17/SC/0168). It was prospectively registered on ISTCRN registry (ISRCTN14279335) in September 2017 and updated in March 2019 to include the addition of inviting participants to this qualitative study. The PCSHOP trial was completed on 16 January 2019. From the 113 randomised, a total of 106 (94%) participants were followed-up at three months: 46/48 ‘BS’ group, 44/48 ‘SF’ group, and 16/17 ‘controls’.

### 2.2. Sampling Procedure and Recruitment

We invited the 44 participants from the ‘SF’ group to join the qualitative study. These participants received a letter invitation for a telephone interview. The invitation letter asked participants to share their experience of taking part in the PCSHOP trial regardless of whether this was good or bad, and encouraged participants to contact the research team for further information. Three participants declined to participate; one due to poor health and two gave no reasons. Interested participants were mailed a participant information sheet and a telephone interview was arranged. Recruitment continued until sufficient data were obtained to address the research aims. Interviews for this qualitative study commenced on 27 February 2019 and finished on 29 March 2019. All interviewed participants were offered £20 for participating.

### 2.3. Interviews

Interviews were facilitated by a female research assistant (CL) who had experience in behavioural interventions and in conducting semi-structured interviews related to diet and nutrition. Prior to the interviews, CL was trained in qualitative interviewing and the PCSHOP intervention, which involved observing the three-month follow-up visit of three participants. The interviewer had not interacted with the participants. Participants knew CL was a member of the PCSHOP trial team.

The study team developed a semi-structured topic guide based on the PCSHOP intervention components and experience of the research conducted (see Supplementary Material S1). The topic guide was piloted with an external member of the research team. The guide consisted of 10 open-ended questions prompting the participants’ to describe their experience of the intervention in chronological order: (1) experience of the brief advice session provided by the nurse, and (2) experience of the shopping feedback. The interviewer probed responses to elicit aspects of the intervention that were perceived as useful or not.

To minimise participant burden, all interviews took place once over the telephone at a time and place convenient for the participant. All participants gave informed consent, and the interview was recorded and transcribed verbatim. Interviews lasted on average 41 min (range = 22–69 min). No new data emerged after eighteen interviews, but we interviewed six more participants that confirmed that no new concepts were emerging.

### 2.4. Data Collection and Analysis

Each audio-recorded interview was independently transcribed and checked by the interviewer for accuracy against the recording. The interviewer then anonymised and encrypted each transcript. The analytical strategy aimed to understand participants’ subjective experiences of the trial. The end goal was to understand the acceptability and usefulness of using brief support and personalised shopping feedback, and the factors that influenced participants’ decision-making when purchasing food.

We followed a descriptive qualitative analysis aiming to learn about and to describe phenomena about which little is known [17]. We did not intend to generate new theories or concepts, but rather identify how participants valued brief support delivered in routine primary care and the personalised shopping feedback. Our ontological position was relativism, and our epistemology was rooted in subjectivism.

We used a thematic analysis to code, categorise, identify, and describe patterns in our data and did not generate any new theories [18,19]. First, we developed an initial coding frame based on the trial’s aim and intervention components, which was discussed with the chief investigator after each interview. The interviewer then coded each transcript line-by-line to further develop the data-driven coding frame. The interviewer kept a record of thoughts on reading each transcript and a reflexivity log of personal beliefs and experiences. This record was discussed with the chief investigator throughout the process of data coding. The One Sheet of Paper (OSOP) technique was used to inductively group codes into broader categories [20]. Finally, categories were summarised to produce top-level analytic themes. Thematic groupings were discussed with all authors to reach a consensus agreement. All data was managed in NVivo 12 software [21]. Transcripts were not returned to the participants and they did not provide feedback on the findings. Selected quotations are presented below, and personal and brand names have been anonymised.

## 3. Results

### 3.1. Participant Characteristics

**Figure 1:**
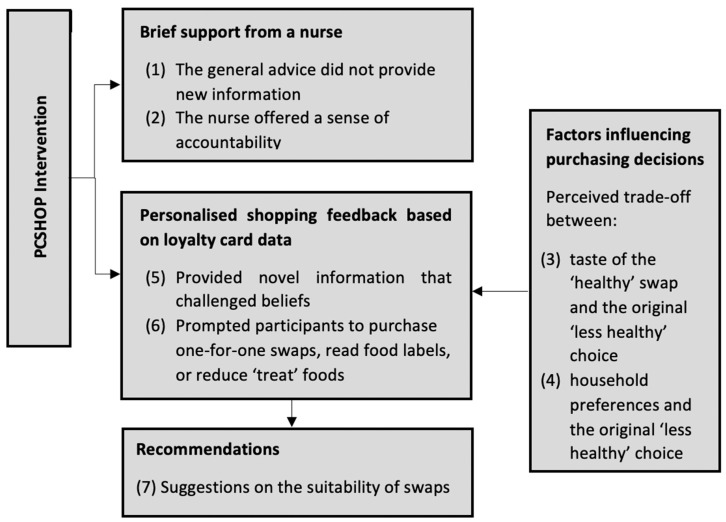
PCSHOP intervention components and related themes.

Twenty-four of the 44 people invited were interviewed. The mean age was 63 years (standard deviation [SD] 12) and 16 were women. The participants had similar baseline demographics to the participants in the main PCSHOP trial (see Table 1). Most participants visited the participating supermarket at least once a week and purchased foods for themselves and one other person, usually their spouse. The thematic analysis identified four key themes related to our aim, which are presented according to the intervention components (see Figure 1).

### 3.2. Brief Support from a Healthcare Professional

#### 3.2.1. Theme 1. Novelty of the General Advice

Participants reported the brief advice did not provide any new information. They highlighted that their family doctor had already advised them on ways to make dietary and other behavioural changes to reduce their cholesterol prior to joining the trial.

[I had] *the high sort of cholesterol results quite some time before, and sort of a vague discussion with the doctor*

(JM, female)

Some believed they had already made the necessary dietary changes either as a result of the individual advice or more general population health messaging and felt there was little scope to change much else.

*There wasn’t too much that we could change because I think we do fairly well with what we eat*

(CJ, female)

In other instances, participants reported that their family doctor had advised their LDL cholesterol, although raised, was not a concern.

*Well I wasn’t too worried about the cholesterol because the doctor had said it wasn’t a problem ‘cos it was only just over*

(HC, female)

*I had a letter asking me to take part in the study, and I believe it was because it was as a result of a cholesterol blood test, which I was told by my [family doctor] at the time I had nothing to worry about*

(AL, female)

#### 3.2.2. Theme 2. Accountability to the HCP

Although participants did not find the advice itself novel, they did report meeting with a nurse provided a sense of accountability, which motivated them to stay focused on the healthfulness of their diet.

*It’s like an exam at school. So you were thinking ‘oh, I’d better do this, before my exam’ … because I’m going to have my cholesterol checked. Whereas sometimes it’s only done once a year*

(CS, female)

*To stay motivated … perhaps if had been any longer… I think perhaps interest would fade a bit without having [the checks]*

(JM, female)

Some participants reported trying harder to reduce their saturated fat intake after the 3-month follow-up visit results showed their cholesterol had not reduced from baseline.

*Since the study stopped I’ve actually gone further and my wife and I both eat I would say more healthily now than we did even during the study*

(JB, male)

### 3.3. Personalised Shopping Feedback Based on Loyalty card Data

#### 3.3.1. Theme 3. Novel Information Challenging Previously Held Beliefs

Overall, participants reported a more positive experience of the personalised shopping feedback compared with brief advice.

*I think the, for me personally, the shopping reports were more helpful than the time spent with healthcare professional, because she just skimmed over the basics, and I knew the basics*

(VV, female)

Participants found the data on the saturated fat content of their food shopping surprising because it contradicted existing beliefs that they were already eating foods low in saturated fat.

*Yeah, it was an eye-opener. It certainly was. Because some things I just thought ‘oh, it’s nothing’, but no it is … You know, even if it’s like one is biscuit a day or something [laughs], you know, it’s still counting*

(MC, female)

Participants reported that this novel information prompted them to act on the shopping reports by purchasing the one-for-one swaps to reduce their saturated fat intake further than they thought possible or necessary, or to reduce the number of ‘treat’ foods.

*I sort of have a few sort of cheat treats that I probably shouldn’t eat, but I’m working on the assumption that as long as overall everything is lower then it’ll be okay*

(JB, male)

Similarly, participants found the top five major contributors to saturated fat helpful, and some participants reported that it prompted them to start paying closer attention to food labels.

*I do look at [food labels] now, which before, I did get very lax. I do look at them all now … it was your information that you were giving me*

(EV, female)

#### 3.3.2. Theme 4. Acceptability of the One-for-One Healthier Swaps

Most participants reported trying the suggested swaps at least once. Some perceived a trade-off between the taste and healthfulness of the food item. These participants reported the need to compromise by either accepting the less palatable item for the benefit of their health, or adapting their behaviours by purchasing the original product but reducing their portion size or consumption frequency.

*I could change to the healthier version, and enjoy eating them. But in one or two instances I decided I would stick with my likeable product, but not eat as much of it*

(VV, female)

Participants also perceived a trade-off between the household preferences and healthfulness of the food. Some participants compromised by buying two different versions of the same product—one for themselves and one for other household members.

*I have made these adjustments for me, but haven’t necessarily you know made the same adjustments for the rest of the family*

(TA, female)

Most participants shopped for at least one other person. They suggested a mechanism that captured food purchases bought for their own individual consumption, which would likely boost capability to engage with the intervention by making the swap suggestions more relevant.

*The suggestions that I should be swapping were very often the things that weren’t mine [laughs], weren’t, you know, weren’t things I was going to eat at home. They were things that the—I was either getting them in for the grandchildren or for my husband … there had to be something else, didn’t there, another step somewhere to identify what was my shopping, I suppose*

(JM, female)

Others wanted more swap suggestions. This was particularly true for participants who shared their loyalty cards and received feedback on foods purchased by other members of the household.

*Oh, you’d almost like more of it, so there’d probably be about three or four swaps on there, you know if I’ve got a hundred items, it was only, you know 3% or 4% of what I’d actually shopped*

(SK, female)

In some instances, the collaborating supermarket did not stock the swap suggestion. Some participants reported using the advice from nurse to make their own swap in the same supermarket or using food labels.

*I did struggle sometimes to make the swaps ‘cos they hadn’t got what was available. So, what I ended up doing was I started paying a lot more attention to the back of labels, and reading how much saturated fat stuff was, and going for the lowest I could find in each sort of product category*

(JB, male)

Lastly, most participants shopped in more than one supermarket and reported that doing so meant that they could not take the swap suggestion, which was not available outside their ‘home’ supermarket. They suggested a method to capture food purchases across various supermarkets would have made opportunities to spot and purchase healthier foods more realistic.

*Because I couldn’t find some of those items originally, I just ended up buying you know normal full-fat cheese, when obviously I went with the intention of pursuing the right thing*

(TA, female)

## 4. Discussion

In this qualitative study, we report participants’ experiences of the PCSHOP trial where they received brief advice from a nurse in the primary care practice, together with personalised feedback on food purchases based on nutritional analysis of loyalty card data from the UK’s largest grocery store. Despite the brief advice from the nurse not providing any new information, participants reported contact with a nurse provided them with a sense of accountability. On the other hand, loyalty card data presented a well-received opportunity to provide regular personalised feedback on shopping behaviour that revealed novel insights on the healthfulness of food purchases. Although participants reported this shopping advice encouraged them towards some positive dietary changes, like an attempt to try a swap suggestion, taste preferences of the participant or other household members remained a barrier to changing food shopping behaviour.

Generally, the brief advice did not provide any new information to participants either because they felt they were already making necessary changes to their diet or did not perceive they were at risk of CVD. It is possible participants may not have had other risk factors for CVD to warrant any further attention from their family doctor or treatment with statin medication. Hence, it is possible that some participants may have been unconvinced of the personal benefit of reducing saturated fat intake based on the brief advice. However, there was some evidence that participants were ‘primed’ by this advice to alter their behaviour, and this may have been supported by the personalised feedback in their shopping reports. Nonetheless, despite reservations about the content of the session with the nurse, participants did feel accountability was an important motivating factor, and for this reason wanted more frequent contact with a HCP beyond their health check that is currently offered by the National Health Service for people aged 40 to 64 years in the UK.

These results help inform the growing interest in using loyalty card data to provide feedback on dietary behaviours. Observational studies have used it to monitor patterns in supermarket food purchases [22], and high-street medications offered by retailers [23]. Besides PCSHOP, to our knowledge only one other previous RCT utilised supermarket loyalty card data in a behavioural intervention to change food purchasing behaviour [12]. In that RCT, participants could access a website to receive personalised feedback and previous food purchases specific to ready meals and pizzas and set individual goals by using the traffic light system to purchase healthier food alternatives. The authors reported no evidence that the intervention changed purchasing or motivation-related factors that would support behaviour change. However, our qualitative study suggests the personalised shopping feedback provided novel information on saturated fat intake that challenged participants’ pre-existing beliefs, namely what foods were high in saturated fat and the healthfulness of their diet. This feedback could have motivated participants to make changes beyond what they thought capable or necessary, and the one-to-one swap suggestions helped them spot opportunities to buy lower saturated foods. Overall, our findings point towards using objective data on food purchases as a potentially useful tool in encouraging participants’ subjective decision-making process towards healthier food purchasing.

Using loyalty card data makes assumptions about the participating individual’s consumption based on household level purchase data. In our study, most participants shared responsibility for household food purchases with their spouse, which meant the feedback, although direct, was not personal to the participant. Similar difficulties in balancing the needs of other household members have been reported in other qualitative studies [24,25]. A more obvious challenge faced by future trials is that participants distribute their purchases over several grocery store retailers, meaning they did not always spot an opportunity for healthier food purchases. Most grocery stores use customer loyalty card programmes [26,27], and in the UK almost 90% of the public belong to at least one loyalty card programme [28]. This presents the opportunity to link multiple loyalty card data sources with innovative digital technologies to deliver accessible and tailored behavioural support in real time as a public health initiative. The advantage of using loyalty card data is that it can be automated and delivered at relatively low-cost to large numbers of people.

In the PCSHOP trial, one in four swaps appeared in the subsequent purchase record during the intervention period, deemed ‘accepted swaps’ [15]. In our study, findings suggest a perceived trade-off between the palatability of the suggested ‘healthy’ swap and the original ‘less healthy’ choice. Participants may have placed higher value on instant gratification (i.e., preferred food taste, being amenable to household preferences) and lower value on perceived benefits achieved at some point in the future (i.e., reduced CVD risk) [29]. Qualitative studies exploring key influences on high saturated fat food purchases report taste, price, and other nutrients like sugar and salt as important though this evidence is based on hypothetical scenarios [30]. Whilst little can be done to change taste perceptions, machine learning algorithms could, over time, offer more suitable swaps that are accepted by people, and there is some evidence that if eating habits change then preferences will follow [31]. On price, no participant reported it as influencing their decision-making, which may reflect the relatively affluent characteristics of our sample, although the PCSHOP trial reported no change in the overall price of the shopping, as seen in similar studies [11]. Further qualitative interviews in economic and ethnically diverse samples are warranted.

Qualitative studies provide added value to clinical trials by exploring the experiences of participants that could offer insight on how the intervention affected behaviour and the influencing factors to be considered in future trials. The main strength of our study is that we asked participants to reflect on direct experiences rather than proposing hypothetical scenarios, which is likely to improve the validity of our results. We used semi-structured, one-to-one interviews, which allowed flexibility for participants to describe aspects of the experience that were important for them. Another strength is that the interviews were facilitated by an interviewer who was unknown to the participants but who had detailed knowledge of the main PCSHOP trial. The absence of a prior rapport between the interviewer and participants’ likely reduced response bias. Limitations include recruitment bias, which is common in follow-up studies related to diet and health possibly because those who are less successful are usually reluctant to take part in interviews. Researcher bias also cannot be entirely excluded, although the interviewer was trained in qualitative methods and all researchers contributed towards data analysis. Another limitation is that white ethnic groups were over-represented in our sample, as were with higher educational attainment. Although this reflects the demographic profile of the population taking part in the main PCSHOP trial and the broader demographics of Oxfordshire [32], results may not generalise across the UK. Future studies could consider recruiting from the wider population and explore if or how differences in demographic profiles influence abilities to positively engage in feedback from supermarket loyally card data.

## 5. Conclusions

This qualitative study described participants’ experiences of an intervention that offered brief general advice from a nurse plus feedback using supermarket loyalty card data to reduce saturated fat intake. Despite the perception that the nurse advice did not provide any new information to participants, contact with a nurse did offer a sense of accountability that may have primed participants to attempt dietary change. Providing regular and personalised feedback on shopping behaviour, based on supermarket loyalty card data, offered participants revealing insights into their food choices, and this may have encouraged some positive dietary changes. However, established taste preferences of the participant or other household members remain a significant barrier to changing food purchasing behaviours. These findings provide insights into the experience of participants, which can help this type of intervention. Importantly, we have shown that using supermarket loyalty card data in this way is acceptable to customers. As such, this approach offers the opportunity to develop future interventions, in collaboration with grocery store partners, that are automated and scalable, with the potential to deliver population-level health benefits.

## Figures and Tables

**Table 1:** Participant Characteristics.

Participant Characteristics	m (SD) ^b^
Age (years)	63.1 (12.1)
	*n* (%)
**Gender**	
Male	8 (33.3%)
Female	16 (66.6%)
**Educational Level ^a^**	
Up to 4 GCSEs or equivalent	4 (16.7%)
5+ GCSEs, or 1 A Level or equivalent	3 (12.5%)
2+ A Levels or equivalent	4 (16.7%)
Bachelors	4 (16.7%)
Postgraduate	7 (29.2%)
**Ethnicity**	
White (British, Irish or other)	23 (95.7%)
Asian or Asian British	1 (4.2%)
**Smoking Status ^a^**	
Never	15 (62.5%)
Ex-smoker	7 (29.2%)
Current smoker	1 (4.2%)
**Alcohol Consumption**	
Never	2 (8.3%)
Sometimes	10 (41.7%)
Every week	12 (50.0%)
If every week, average units per week, m (SD)	4.9 (8.2)
**Grocery shopping behaviour, how frequently do you visit the participating supermarket**	
≤Once a month	1 (4.2%)
Once a month	1 (4.2%)
Once a fortnight	2 (8.3%)
Once a week	11 (45.8%)
More than once a week	9 (37.5%)
**Grocery shopping behaviour, no. of people in your household**	
1, i.e., just yourself	6 (25%)
2	10 (41.7%)
3	4 (16.7%)
4	2 (8.3%)
≤5	2 (8.3%)
**Grocery shopping behaviour, type of shop**	
Supermarket	24 (100%)
Online	0 (0%)
Corner shop	3 (12.5%)
Green grocers	0 (0%)
Butchers	5 (20.8%)
Other fresh food markets	5 (20.8%)

^a^ missing value(s) ^b^ Results are presented as mean (standard deviation [SD]) or relative frequencies.

## Data Availability

The data that support the findings of this study are available from the corresponding author upon reasonable request.

## Supplementary Materials

The following supplementary material is available https://www.mdpi.com/article/10.3390/nu13041146/s1, Table S1: Topic guide and inquiry logic for semi-structured, telephone interviews.
