# Supplementary material for: Using Supermarket Loyalty Card Data to Provide Personalised Advice to Help Reduce Saturated Fat Intake among Patients with Hypercholesterolemia: A Qualitative Study of Participants’ Experiences
Source: Nutrients. 2021 Mar 31;13(4):1146. doi: 10.3390/nu13041146 (PMC8066863; doi:10.3390/nu13041146)
Supplement: Supplementary file 1 [file nutrients-13-01146-s001.pdf]

Supplementary Material

Table S1. Topic guide and inquiry logic for semi-structured, telephone interviews.

Intervention Component	Question	Example Prompts
**Introduction**	What's your understanding about the effects fat can have on your health?	How do you think the fat in your food might affect your health?<br><br>How do you think about the cholesterol in your blood and its effects on your body?
**Brief Advice Session**		
Discussion of cardiovascular disease risk and motivational support	What did you think about the advice you received from the nurse?	What do you remember the nurse discussing about your recent blood results?<br><br>Did you feel there were important issues that weren't discussed in this session? Give examples.
	Did you try to make any changes to your diet after speaking with the nurse?	In what ways did you think differently about your food when you went shopping?<br><br>How encouraged were you to start changing your diet?<br><br>If you didn't change anything, what do you think is the reason for that?
British Heart Foundation Leaflet	What did you think about the British Heart Foundation leaflet?	How did it help your understanding about fat in your diet?<br><br>How motivated were you to act differently e.g. begin reading food labels, buying different foods?
	Is there any other support your nurse or GP could provide, which you feel would help you to improve your diet?	Did you get advice from anyone or anywhere else? If yes, how helpful was that advice?
**Personalised Feedback on Shopping Report**		
Summary of % SFA in previous food purchases and top 3 foods contributing to SFA	What did you think about the shopping reports?	How did you feel about us looking at your food shopping?<br><br>Did you find the feedback helpful?
	And what did you think about the healthier swaps suggested?	Did you try the swaps?<br><br>Have you stuck to the swaps?<br><br>In what ways did you make any other swaps not suggested in the report?<br><br>Were the swaps reasonable in price, taste, appearance or brand prevent or encourage you to change your shopping?

**Contextual Factors**	Was there anything else that influenced your food shopping?	What other information would you like to have at hand in order to help you improve your food choices?<br><br>Do you feel anyone else in your household affected your shopping choices?<br><br>Is anyone else in your household using the swaps suggestions?
**Future Suggestions**	Imagine we ran this study again. How would you feel about health advice coming from someone else other than your GP, for example a supermarket pharmacist?	
**Close**	**Lastly, are there any final thoughts or experiences about the study that you would like to share?**	
